# Retraction Note: Molecular Phylogeny and Revision of Copepod Orders (Crustacea: Copepoda)

**DOI:** 10.1038/s41598-020-74404-2

**Published:** 2020-10-14

**Authors:** Sahar Khodami, J. Vaun McArthur, Leocadio Blanco-Bercial, Pedro Martinez Arbizu

**Affiliations:** 1 Senckenberg Am Meer, German Center for Marine Biodiversity Research, Südstrand 44, 26382 Wilhelmshaven, Germany; 2 Savannah River Ecology Laboratory, Aiken, SC USA; 3 Bermuda Institute of Ocean Sciences (BIOS), Hamilton St. Georges, Bermuda

Retraction of: *Scientific Reports* 10.1038/s41598-017-06656-4, published online 22 August 2017

The Authors have retracted this Article.

The alignment provided in the supplementary material and uploaded to TreeBASE^1^ is not the one used to construct the tree of copepod orders reported in the Article. Unfortunately we did not retain a copy of the original alignment. Additionally, 9 out of 492 fragments submitted to GenBank were mislabelled. As a result, the published tree of copepod orders cannot be reproduced with the provided alignment, and some of the conclusions reported in the paper cannot be recapitulated.

Sahar Khodami, Leocadio Blanco-Bercial & Pedro Martinez Arbizu agree with this retraction. J. Vaun McArthur could not be reached.
